# Morphological, Molecular, and Growth Characteristics of a Cryptic Species, *Strombidium parasulcatum* n. sp. (Alveolata: Ciliophora: Oligotrichida)

**DOI:** 10.3389/fmicb.2021.770768

**Published:** 2022-02-10

**Authors:** Sheng-Fang Tsai, Meng-Lun Lee, Kuo-Ping Chiang

**Affiliations:** ^1^Institute of Marine Environment and Ecology, National Taiwan Ocean University, Keelung, Taiwan; ^2^Center of Excellence for the Oceans, National Taiwan Ocean University, Keelung, Taiwan

**Keywords:** ciliary pattern, protozoa, phylogeny, *Strombidiidae*, taxonomy

## Abstract

A new marine planktonic ciliate from Taiwan, *Strombidium parasulcatum* sp. n., is described based on live observations, protargol staining, and molecular data. Its morphological characters are similar to those of *Strombidium sulcatum* Claparède and Lachmann, 1859 sensu Song et al., 2000 but differ from *S. sulcatum* sensu Fauré-Fremiet, 1912 and sensu Fauré-Fremiet and Ganier, 1970 by several morphological characters. The 18S rRNA gene sequences of the two forms display 76 base pair differences (about 5%), indicating that they should be considered separate species. The highest observed specific growth rates of *S. parasulcatum* in culture were 1.79 day^–1^ over 1 day and 1.52 day^–1^ over 2 days, both starting at day 5. Bacteria appear to be an important food resource for the cultivation of this medium-sized oligotrich ciliate. This and other recent studies suggest that cryptic species may be common in the genus *Strombidium*, and an integrative approach including morphological, ecological, and molecular data should be used to address this question.

## Introduction

The oligotrich (s. l.) ciliates are a dominant group in the marine microzooplankton. They serve as an effective link between the microbial loop and the grazing food chain due to their high abundance and growth rate (Maeda and Carey, 1985; Maeda, 1986; Fileman and Leakey, 2005; Bojanić et al., 2006; Pierce and Turner, 1992; Agatha, 2011a). Knowledge of their functional diversity is important for understanding their roles in nature (Mitra et al., 2016). With regard to their taxonomic diversity, observations of specimens *in vivo* and following silver staining are regarded as essential for accurate species identification and circumscription (Warren et al., 2017).

Currently, the number of valid oligotrich (s. str.) species amounts to about 120 (Agatha and Strüder-Kypke, 2014), but recent studies revealed that numerous cryptic species in oligotrichs exist, suggesting that their biodiversity is greater than previously assumed and highlighting the need for further investigations of their taxonomy (McManus et al., 2010; Agatha, 2011b). Although live observations are indispensable for a good description of oligotrich species, they are unfortunately lacking in many publications. Due to the phenotypic plasticity and lack of reliable morphological features for many oligotrich ciliates, it is often difficult or even impossible to separate cryptic species based on morphology alone (Katz et al., 2005; Liu et al., 2017). Hence, the small subunit (18S) rRNA gene is increasingly being sequenced to supplement morphological observations. The recent treatments of new or poorly known taxa suggest that integrating molecular and morphological data provides a reliable method to distinguish similar species (Modeo et al., 2003; Agatha et al., 2005; Agatha and Strüder-Kypke, 2007; Strüder-Kypke and Lynn, 2008; Tsai et al., 2008, 2010, 2015; Liu et al., 2009, 2011a,b,^2015^, ^2017^; Kim et al., 2010; McManus et al., 2010; Xu et al., 2011; Chen et al., 2013; Qu et al., 2015; Song et al., 2015a,b).

The species in the genus *Strombidium*, with a horizontal girdle kinety, a longitudinal ventral kinety, and the oral primordium posterior to the girdle kinety, exhibit great biodiversity (Agatha and Strüder-Kypke, 2014). Phylogenetic analyses based on genetic data have revealed the non-monophyly of the genus *Strombidium*, producing highly variable tree topologies with very low support values (Gao et al., 2009; Zhang et al., 2010; Li et al., 2013; Agatha and Strüder-Kypke, 2014). The phylogenetic relationships of members of this genus demonstrates the need for more gene sequences of properly identified oligotrich species. Recent studies also suggest that cryptic species in the genus *Strombidium* may be common, highlighting the need to conduct investigations using multiple taxonomic technologies (McManus et al., 2010).

*Strombidium parasulcatum* n. sp., which is very similar in morphological characters to *Strombidium sulcatum* Claparède and Lachmann, 1859 (sensu Song et al., 2000), is here described based on live observation, protargol-impregnated material, and 18S rRNA gene sequences of specimens collected from the northeastern coastal waters of Taiwan in 2011. In addition, we present its growth rates and report that bacteria appear to be an important food resource for this oligotrich ciliate. By combining morphological, molecular, and ecological data, we were able to identify and circumscribe this cryptic species and clarify its phylogeny position.

## Materials and Methods

### Sample Collection, Observation, and Identification

Plankton samples were taken with a 20-μm mesh plankton net from the coastal waters of northeastern Taiwan (25° 08′ 30″ N; 121° 47′ 42″ E) on September 8, 2011. Water temperature and salinity were about 27°C and 33 psu, respectively.

The behavior of the specimens was observed in a Petri dish (about 9 cm across; water depth about 1 cm) under a dissecting microscope (40–70×; Nikon, SMZ-U). After isolation, specimens were observed, using bright field and differential interference contrast microscopy (Nikon, OPTIPHOT-2). Ten living cells were studied at magnifications of 125–1,250×. Illustrations of live specimens were made from free-hand drawings and photomicrographs. Protargol impregnation followed the protocol of Song and Wilbert (1995). Protargol-stained cells were drawn, using a camera lucida (Nikon, Y-IDT). Terminology and systematics follows Agatha and Riedel-Lorjé (2006).

### Culture

The monoclonal *S. parasulcatum* n. sp. was cultured in Petri dishes with bacteria as prey in the water column at 25°C on a 12:12 h light/dark cycle for 45 days. The salinity of the culture medium was 33 psu, matching the ambient salinity. To observe changes in the abundance of ciliates and bacteria during the culture period, a source culture in a 500-ml polycarbonate (PC) bottle was prepared by putting 500 ml of 0.2-μm filtered sea water into the bottle along with five grains of rice to raise bacteria. Two days later, when bacteria had reached a sufficient concentration, 500 cells of *S. parasulcatum* n. sp. were added to the bottle. Subsamples (10 ml) were then collected in the noon at 1-day intervals after gently but thoroughly mixing the PC bottle.

To count bacteria, subsamples of 1 ml culture sea water each were fixed immediately by adding glutaraldehyde to a final concentration of 1% and then were filtered onto 0.2-μm black Nuclepore filters under low pressure (< 100 mm Hg) with a 0.45-μm pore size Millipore filter used as a backing pad to obtain an even distribution of cells. Bacteria left on the filter membranes were then stained with 4′6-diamidino-2-phenylindole (DAPI) at a final concentration of 1 μg ml^–1^ (Porter and Feig, 1980) and counted under an epifluorescence microscope at 1,000× magnification (Nikon Optiphot-2, Japan). For the enumeration of *S. parasulcatum* n. sp., a 9-ml subsample of the culture was fixed with Lugol’s solution to a final concentration of 5%, concentrated following the Utermöhl method (Hasle, 1978), and counted under an inverted microscope (Nikon-TMD 300, Japan) at 200× or 400× magnifications.

### Extraction, Amplification, and Sequencing of rRNA Gene

DNA extraction was done according to Tsai et al. (2008, 2010). Polymerase chain reaction (PCR) was performed in a buffer containing 3 mM MgCl_2_, 0.25 mM dNTP, 10^–3^ mM forward (5′-AAC CTG GTT GAT CCT GCC AGT-3′) and reverse (5′-TCC TTC TGC AGG TTC ACC TAC-3′) primers (Medlin et al., 1988), and SuperTaq polymerase (HT Biotechnology, Cambridge, United Kingdom). PCR involved one cycle of 5 min at 95°C; 35 cycles of 30 s at 95°C, 30 s at 56°C, and 2 min at 72°C; and a final cycle of 10 min at 72°C. The PCR fragments of three clones were ligated into a pGEM-T vector (Promega, Madson, WI, United States) and sequenced by ABI Prism 3730.

### Phylogenetic Analyses

**FIGURE 1:**
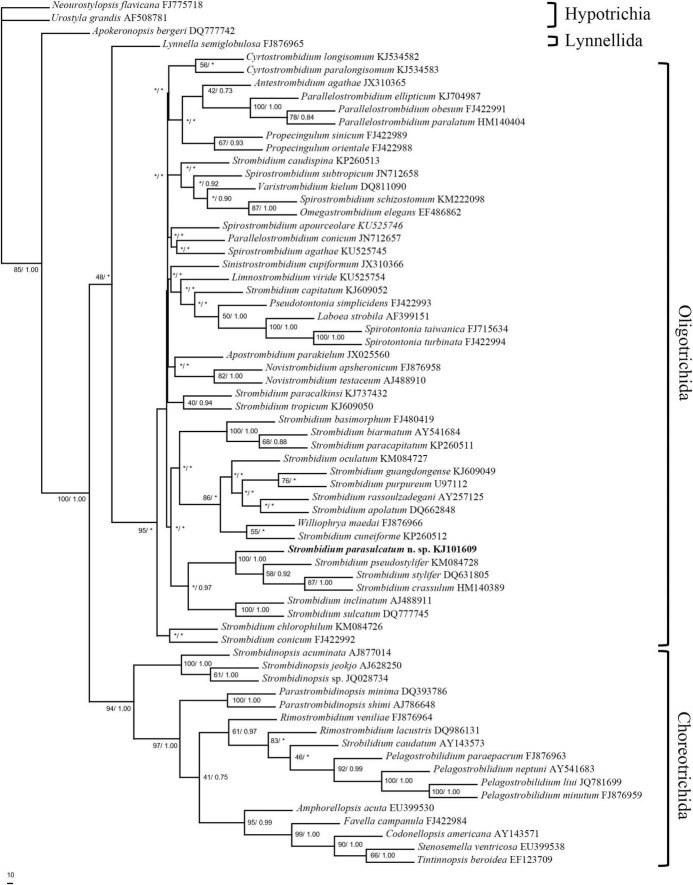
Maximum parsimony analysis tree inferred from 18S rRNA gene sequences of spirotrich ciliates showing the position of *Strombidium parasulcatum* n. sp. Numbers on the branches are support values for the internal nodes followed by the maximum parsimony bootstrap values and Bayesian posterior probabilities (MP/BI). Asterisks mark nodes with support values below 40%. The scale bar corresponds to 10 genetic variation.

All 18S rRNA gene sequences used for phylogenetic analysis were obtained from GenBank/EMBL databases, including 18 choreotrichids, 46 oligotrichids, and 3 hypotrich species as outgroup (see Figure 1 for accession numbers). The alignment of these sequences with that of *S. parasulcatum* was performed, using CLUSTAL W (Thompson et al., 1994). Ambiguous nucleotide positions at the beginnings and ends of fragments were deleted, thus resulting in a data set consisting of 1,293 nucleotide positions. For the phylogenetic analysis, MODELTEST (Posada and Crandall, 1998) was employed to find the model (GTR + Γ model) of DNA substitution that best fit our data. The parameters (nst = 6, rates = γ, *n* = 4, and temperature = 0.2) were implemented into MrBayes ver. 3.1.2 (Huelsenbeck and Ronquist, 2001). A Bayesian Inference analysis (BI) was performed over 4,000,000 generations with two simultaneous and completely independent analyses starting from different random trees. The posterior probability of a phylogeny out of 75,001 trees, approximated via the Markov chain Monte Carlo method with sampling every 50th generation (tree), was computed, with the first 5,003 trees being discarded as burn-in. A maximum parsimony (MP) analysis was performed with PAUP* ver. 4.0b10 (Swofford, 2002).

## Results

### *Strombidium parasulcatum* n. sp.

**FIGURE 2:**
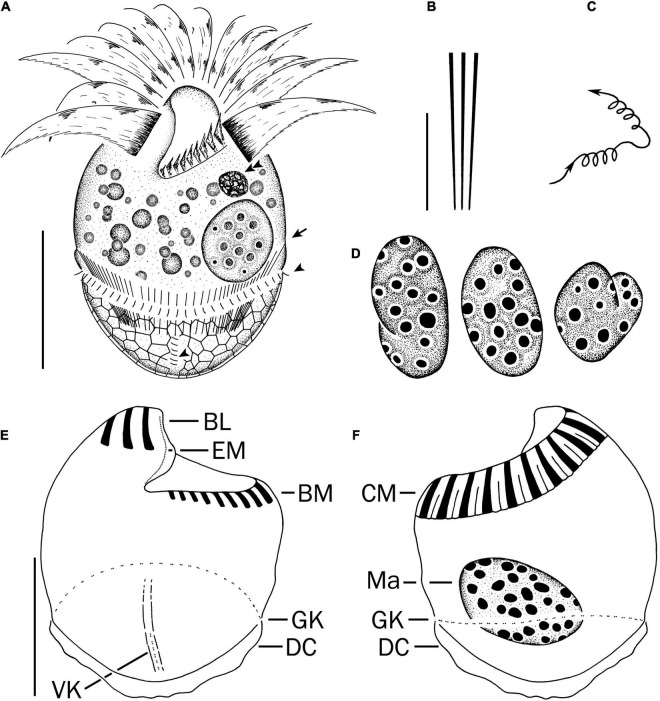
*Strombidium parasulcatum* n. sp. from life **(A–C)** and after protargol impregnation **(D–F)**. **(A)** Ventral view of a representative specimen, with an arrow indicating an extrusome, arrowheads marking the cilia on each left and each anterior basal body in the girdle kinety and ventral kinety, respectively. **(B)** Extrusomes. **(C)** Pattern of locomotion. **(D)** Macronuclei of diverse shapes. **(E,F)** Ventral and dorsal views of the holotype specimen. BL, buccal lip; BM, buccal membranelles; CM, collar membranelles; DC, distended cell surface; EM, endoral membrane; GK, girdle kinety; Ma, macronucleus; VK, ventral kinety. Scale bars = 20 μm **(A,E,F)** and 10 μm **(B)**.

**FIGURE 3:**
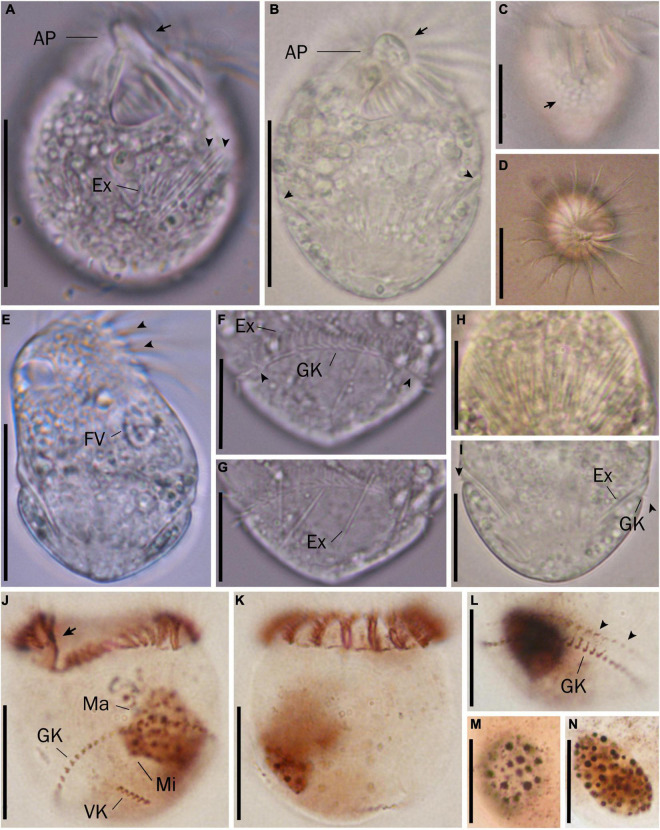
Micrographs of *Strombidium parasulcatum* n. sp. from life **(A–I)** and after protargol impregnation **(J–N)**. **(A)** Ventral view of the shorter specimen to show the apical protrusion (arrow) and the extrusomes (arrowheads). **(B)** Ventral view of a representative specimen to show the apical protrusion (arrow) and the bulge formed by the extrusome attachment sites (arrowheads). **(C)** Hemitheca, composed of polygonal platelets (arrow). **(D)** Apical view showing the apical protrusion and collar membranelles. **(E)** Dorsal view of a deformed specimen showing gaps between neighboring collar membranelles (arrowheads) and food vacuole. **(F)** Cilia of the girdle kinety (arrowheads). **(G)** Extrusomes with rounded anterior and pointed posterior end. **(H)** Extrusomes arranged probably in a single row, posteriorly extending obliquely into cytoplasm. **(I)** Cilia of the girdle kinety (arrowheads). **(J,K)** Ventral and dorsal views of the holotype specimen, with an arrow marking the endoral membrane. **(L)** Dorsal view showing the girdle kinety and the extrusome attachment sites (arrowheads). **(M,N)** Macronuclei of diverse shapes. AP, apical protrusion; Ex, extrusome; FV, food vacuole; GK, girdle kinety; Ma, macronucleus; VK, ventral kinety. Scale bars = 30 μm **(A–E)**, 20 μm **(J,K)**, and 15 μm **(F–I,L–N)**.

Cell size *in vivo* is 40–50 × 30–40 μm, usually 45 × 35 μm; after protargol impregnation, it becomes slightly shorter, viz., 29–41 × 27–40 μm, usually 35 × 34 μm (Table 1). Its shape is slightly variable, generally broadly obconical to ellipsoidal; circular in cross-section, widest in equatorial area, and slightly tapered in posterior half, with posterior end broadly rounded to bluntly pointed (Figures 2A,E,F, 3A,B,E). Apical protrusion is about 5 μm high *in vivo* (Figures 2A, 3A,B,D), sometimes undetectable (Figure 3C). Buccal cavity is shallow but wide, obliquely extending posteriorly to 25% of cell length (Figures 2A,E and 3A,B,J), 15 μm in width. There is buccal lip on the right side of the oral cavity, forming small longitudinal ridge ventrally (Figure 2A). Macronucleus in left cell half is 10–28 × 8–14 μm in size after protargol staining, variable in shape from spherical to bilobed shape, typically ellipsoidal, containing numerous nucleoli 1–2 μm in diameter (Figures 2A,D,F, 3J–N). Neither a contractile vacuole nor the cytopyge is recognized. Cortical platelets (hemitheca) posterior to girdle kinety are polygonal, about 3 μm across (Figures 2A, 3C). Cell surface posterior to girdle kinety is distinctly distended in live and prepared cells, except for the region around ventral kinety where a furrow formed. Extrusomes are prominent *in vivo*, acicular, about 15 × 0.5 μm in size, with rounded anterior and pointed posterior end, closely bundled posterior to the girdle kinety, attached probably in a single row to shallow bulge ∼4 μm anterior to girdle kinety, posteriorly extending obliquely into cytoplasm (Figures 2A,B, 3A,F–H); attachment sites are visible as dotted stripe in protargol-impregnated cells (Figure 3L) but sometimes not observed in over-bleached specimens (Figure 3J). Oral primordium is posterior to girdle kinety and left of ventral kinety (Supplementary Figure 1). Cytoplasm is colorless, full of lipid droplets about 3 μm in diameter (Figures 3A,B). Food vacuoles contain diatoms when cells are collected in the sampling site. They rotate about the main cell axis while swimming (Figure 2C). Cell is bursting easily when water temperature increases or when in contact with water surface.

**TABLE 1:** Meristic and morphometric characterization of *Strombidium parasulcatum* n. sp. based on protargol-impregnated specimens.

Character	$\bar{\text{x}}$	M	SD	SE	CV	Min	Max	n
Cell, length	35.4	36.0	3.7	0.7	0.10	29.0	41.0	25
Cell, width	33.8	34.0	4.0	0.8	0.12	27.0	40.0	25
Cell length/width, ratio	1.1	1.0	0.1	0.0	0.07	0.9	1.2	25
Anterior cell end to buccal vertex, distance	12.2	12.0	2.2	0.5	0.18	8.0	18.0	21
Buccal cavity, width	15.0	14.0	2.8	0.6	0.19	11.0	20.0	25
Cell length/distance anterior cell end to buccal vertex, ratio	3.0	3.0	0.7	0.1	0.22	2.1	5.0	21
Anterior cell end to macronucleus, distance	14.1	14.0	2.8	0.6	0.20	10.	21.0	25
Anterior cell end to girdle kinety, distance	23.1	24.0	3.3	0.7	0.14	16.0	28.0	25
Macronucleus, length	18.9	18.0	4.1	0.8	0.22	10.0	28.0	25
Macronucleus, width	10.6	10.0	1.5	0.3	0.14	8.0	14.0	25
Micronucleus, number	1.0	1.0	0.0	0.0	0.00	1.0	1.0	9
Micronucleus, diameter	3.4	3.0	0.7	0.2	0.21	3.0	5.0	9
Adoral zone of membranelles, outer diameter	27.9	28.0	2.6	0.5	0.09	22.0	32.0	25
Collar membranelles, number	14.5	14.0	0.8	0.2	0.05	13.0	16.0	25
Collar membranelles, distance between bases	2.1	2.0	0.3	0.1	0.16	2.0	3.0	25
Buccal membranelles, number	8.6	9.0	1.2	0.3	0.13	7.0	10.0	19
Girdle kinety, number of dikinetids	55.1	56.0	4.2	1.0	0.08	48.0	64.0	19
Ventral kinety, length	6.2	6.0	1.1	0.3	0.17	4.0	8.0	15
Ventral kinety, number of dikinetids	6.9	7.0	1.2	0.3	0.18	4.0	8.0	17

*Measurements in micrometers.*

*CV, coefficient of variation; M, median; Max, maximum; Min, minimum; n, number of individuals investigated; SD, standard deviation; SE, standard error of arithmetic mean; $\bar{x}$, arithmetic mean.*

Somatic cilia are 2–3 μm long *in vivo*, arranged in a girdle kinety and ventral kinety. Girdle kinety, positioned in posterior one-third (∼65%), composed of 48–64 horizontally orientated, continuously arranged dikinetids, each has associated a cilium only with its left basal body (Figures 2A,E,F, 3F,J,K). Ventral kinety, ∼10 μm posterior to girdle kinety, extends meridionally in argyrophilic furrow on ventral side occupying posterior one-fourth of cell length, composed of four to eight meridionally orientated dikinetids, each with its anterior basal body ciliated (Figures 2A,E, 3J).

Oral apparatus occupies anterior cell portion. Adoral zone of membranelles surrounds apical protrusion, about 28 μm across, terminates about 30% posterior to anterior end of cell, divided into a collar and buccal zone portion (Figures 2E,F, 3J,K). The collar portion is distinctly slanted leftwards and decreases in height from right to left in protargol-stained specimens, composed of 15 membranelles, on average, continuous with buccal portion comprising nine membranelles. Collar membranelles are composed of three rows of basal bodies, 8 μm in length, with cilia up to 25 μm long, orientated perpendicular to the main axis in swimming specimens (Figures 2A, 3D). Argentophilic fibers present between and parallel to collar membranelles (Figure 2F). Collar membranelles are about 2 μm apart (Figure 3E). Buccal membranelles are composed of three rows of basal bodies, except for the proximal most one with two rows of basal bodies, decreasing in width from 5 μm in distal portion to about 2 μm in proximal zone portion (Figures 2A,E, 3A,B,J). The endoral membrane is composed of a single row of basal bodies (probably with stichomonad structure) and extends longitudinally on the inner wall of the buccal lip (Figure 2E).

### Cultivation

During a 45-day cultivation, the average and minimum concentrations of bacteria were 1.5 × 10^7^ and 8.6 × 10^6^ cells ml^–1^, respectively (Supplementary Figure 2A). The growth curve of *S. parasulcatum* n. sp. performed a maximum growth rate at day 5 shown in dashed line (Supplementary Figure 2B). *Strombidium parasulcatum* n. sp. showed its highest 1- and 2-day specific growth rates, 1.79 and 1.52 day^–1^, respectively at day 5; the growth rate decreased thereafter, even falling below 0 day^–1^ (Supplementary Figure 2C).

### 18S rRNA Gene Sequence and Phylogenetic Analyses

The 18S rRNA gene sequence of *S. parasulcatum* n. sp. (GenBank accession no. KJ101609) was 1,729 nucleotides long. The sequences of the three clones were identical. A pairwise distance matrix of six morphologically similar *Strombidium* species was generated, using the p-distance method (Table 2). Nucleotide pairwise distances ranged from 0.0000 (the variation between *S. sulcatum* and *Strombidium inclinatum*) to 0.0459 (the variation between *S. parasulcatum* and *S. sulcatum* and *S. inclinatum*, respectively). With the differences of 76 bp, it was 95% similar to both of *S. sulcatum* (DQ777745) sampled by Gao et al. (2009) in Qingdao, China, and *S. inclinatum* (AJ488911) sampled by Modeo et al. (2003) in Leghorn, Italy. The sequences similarities between *S. parasulcatum* and *Strombidium pseudostylifer*/*Strombidium stylifer*/*Strombidium crassulum* were all 98%, with 28 bp difference.

**TABLE 2:** Estimates of evolutionary distance between 18S rRNA gene sequences of six *Strombidium* species.

	*S. pseudostylifer*	*S. stylifer*	*S. crassulum*	*S. parasulcatum*	*S. inclinatum*	*S. sulcatum*
*S. pseudostylifer*		8	10	28	62	62
*S. stylifer*	0.0048		2	28	58	58
*S. crassulum*	0.0060	0.0012		28	58	58
*S. parasulcatum*	0.0169	0.0169	0.0169		76	76
*S. inclinatum*	0.0375	0.0350	0.0351	0.0459		0
*S. sulcatum*	0.0375	0.0350	0.0351	0.0459	0.0000	

*The pair distance (lower triangle) and base pairs differences of 18S rRNA gene between sequences (upper triangle) are shown. There were a total of 1,659 positions in the final dataset. Evolutionary analyses were conducted in MEGA X, using the p-distance model.*

The topologies inferred, using BI and MP, were basically congruent, and only the MP tree is shown (Figure 1). The new species formed a clade with *S. crassulum*, *S. pseudostylifer*, and *S. stylifer* (MP, 100%; BI, 1.00) that was sister to the clade including *S. sulcatum* and *S. inclinatum* (MP, 36.5%; BI, 0.97).

## Discussion

*Strombidium sulcatum* was first observed by Claparède and Lachmann (1859). In a revision of the species, Montagnes et al. (1990) proposed that the works of Fauré-Fremiet and colleagues collectively characterized *S. sulcatum* and diagnosed the species according to the descriptions of Fauré-Fremiet (1912) and Fauré-Fremiet and Ganier (1970).

Song et al. (2000) found that a detailed redescription concerning the morphology *in vivo* and infraciliature of *S. sulcatum* is still necessary because of its high variability in many respects based on the observation of a population from Yellow Sea and suggested an improved diagnosis. Later, its 18S rRNA gene sequences were extracted accompanied with the identifications investigated *in vivo* and impregnated with protargol (Zhang et al., 2010).

With the improved knowledge of strombidiid structure (Montagnes and Lynn, 1991; Foissner, 1994) and the ready availability of better microscopes, Granda and Montagnes (2003) re-examined Fauré-Fremiet’s silver-stained material collected from the northeastern Atlantic Ocean, which morphologically agree with those presented by Fauré-Fremiet (1912) and Fauré-Fremiet and Ganier (1970) (slides made in the 1950s and 1960s), deposited the type material from Fauré-Fremiet’s slide, and provided authoritative data, annotating their observations with illustrations and micrographs and thereby putting a halt to all discussions about the identity of the species.

Some morphological differences between *S. sulcatum* populations reported from the northeastern Atlantic Ocean (Fauré-Fremiet and Ganier, 1970; Fauré-Fremiet, 1912) and those from the Yellow Sea (Song et al., 2000) were observed in the shape of macronucleus and the transient nature of the protrusion. Granda and Montagnes (2003) suggested not to make a distinction between these two populations until more data are available on this potentially cosmopolitan species (Agatha et al., 2021). Even though these two characters do not have high weight in species level to separate *Strombidium* species, it is not insufficient to make the conclusion that they belong to another, pseudo-cryptic species. However, we take all the similar forms as a complex of *S. sulcatum* and wait for future solution.

### Comparison With Similar Congeners

**FIGURE 4:**
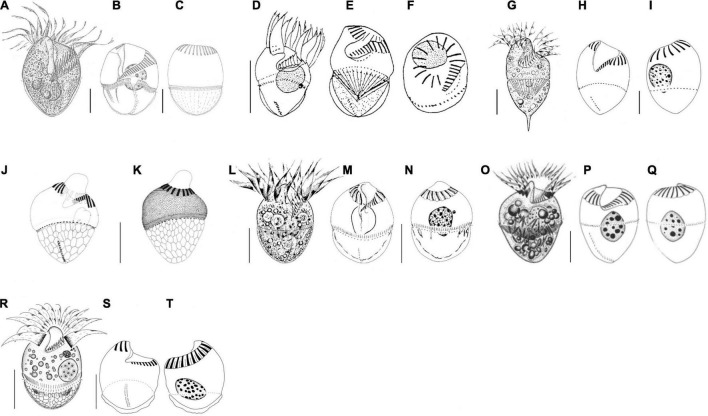
Schematic drawings of seven species compared in Table 3 from life **(A,D,G,L,O,R)** and after protargol impregnation **(B,C,E,F,H–K,M,N,P,Q,S,T)**. **(A–C)** *Strombidium crassulum* (Petz et al., 1995); **(D–F)** *S. inclinatum* (Montagnes et al., 1990); **(G–I)** *S. stylifer* (Song et al., 2015b); **(J,K)** *S. sulcatum* (Granda and Montagnes, 2003); **(L–N)** *S. sulcatum* (Song et al., 2000); **(O–Q)** *S. suzukii* (Xu et al., 2009); **(R–T)** *S. parasulcatum* (this study). Scale bar: 20 μm.

Seven congeners are similar or close to *S. parasulcatum* n. sp., viz., *S. crassulum* Petz et al., 1995, *S. inclinatum* Montagnes et al., 1990, *S. pseudostylifer* Song et al., 2015a, *S. stylifer* Levander, 1894, *S. sulcatum* Claparède and Lachmann, 1859 sensu Granda and Montagnes, 2003, *S. sulcatum* sensu Song et al., 2000, and *Strombidium suzukii* Xu et al., 2009, according to the body shape or the close relationship in the phylogenetic tree (Montagnes et al., 1990; Petz et al., 1995; Song et al., 2000, 2015a,b; Granda and Montagnes, 2003; Xu et al., 2009; Table 3 and Figure 4). Here, we ignored the detailed description of *S. inclinatum* from Modeo et al. (2003) due to the uncertainty of conspecificity for not sampling from the type locality (Baltic Sea) but from the Mediterranean Sea.

**TABLE 3:** Morphometrics comparison among species in the genus *Strombidium*.

Characters	1	2	3	4	5	6	7	8
Cell, length	62 (43–90)	23 (13–30)	73 (55–92)	44 (35–52)	36 (32–41)	30–47	55*	35 (29–41)
Cell, width	54 (35–74)	17 (13–21)	42 (31–54)	28 (24–37)	32 (29–35)	24–41	42*	34 (27–40)
Anterior protrusion, *in vivo*	Inconspicuous	Absent	Present	Present	Present	Present	Conspicuous	Present
Anterior protrusion, stained	Absent	Absent	Absent	Absent	Present	Absent	Present	Absent
Buccal cavity, width	6*	4*	22	17	7*	7*	15*	15 (11–20)
Cortical platelets	Absent	Present	Absent	Absent	Present	Present	Absent	Present
Macronucleus, length	26 (16–37)	9 (6–13)	25 (17–32)	19 (13–26)	14 (12–17)	16*	22*	19 (10–28)
Macronucleus, width	22 (13–28)	9 (6–13)	21 (15–30)	15 (7–25)	12 (9–13)	13*	17*	11 (8–14)
Collar membranelles, number	17 (16–19)	14 (12–15)	15 (14–17)	15 (13–17)	15 (14–15)	13–16	15–16	15 (13–16)
Buccal membranelles, number	15 (13–19)	8 (7–9)	8 (7–9)	8 (5–9)	7 (6–9)	7–9	6–7	9 (7–10)
Girdle kinety, no. of dikinetids	115	44–50*	45 (36–53)	46 (38–58)	44 (37–50)	50 (46–56)	50–66	55 (48–64)
Girdle kinety, gap	Ventral side	Absent	Absent	Absent	Absent	Absent	Absent	Absent
Ventral kinety, no. of dikinetids	11 (9–15)	7–9	23 (17–29)	10 (7–14)	10 (9–11)	5–12	15–17	7 (4–8)
Ventral kinety, length	9*	5*	30	15*	10*	7*	28*	6 (4–8)
Elongate process/spine	Absent	Absent	Present	Present	Absent	Absent	Absent	Absent

*1, Strombidium crassulum (Petz et al., 1995); 2, S. inclinatum (Montagnes et al., 1990); 3, S. pseudostylifer (Song et al., 2015a); 4, S. stylifer (Song et al., 2015b); 5, S. sulcatum (Granda and Montagnes, 2003); 6, S. sulcatum (Song et al., 2000); 7, S. suzukii (Xu et al., 2009); 8, S. parasulcatum (this study). Measurements were made on protargol-impregnated specimens and are presented as means (range). Measurements in μm.*

*Data were obtained from the figures in the original work.

*Strombidium parasulcatum* n. sp. differs from *S. crassulum* in cell size (35 × 34 vs. 62 × 54 μm after protargol staining), buccal cavity width (15 vs. 6 μm), the appearance of cortical platelets (vs. without), lack of an obvious gap in the ventral side of the girdle kinety (vs. with gap), and having fewer collar and buccal membranelles (13–16 vs. 16–19 and 7–10 vs. 13–19, respectively) and fewer dikinetids in the ventral and girdle kinety (4–8 vs. 9–15 and 55 vs. 115, respectively).

*Strombidium parasulcatum* n. sp. can be distinguished from *S. inclinatum* by the cell size (29–41 × 27–40 μm vs. 13–30 × 13–21 μm), the presence of an apical protrusion *in vivo* (vs. absent), buccal cavity width (15 vs. 4 μm), and the numbers of dikinetids in the girdle kinety (48–64 vs. 44–50).

*Strombidium pseudostylifer* and *S. stylifer* are characterized by having an elongate process/spine and can be easily separated from *S. parasulcatum* n. sp. Moreover, *S. parasulcatum* can be separated from *S. pseudostylifer* and *S. stylifer* by the appearance of cortical platelets (vs. without), its shorter ventral kinety (6 μm vs. 30 and 15 μm, respectively), and different numbers of dikinetids in the ventral kineties (4–8 vs. 17–29 and 7–14, respectively).

*Strombidium parasulcatum* n. sp. differs from *S. suzukii* in cell size (35 × 34 vs. 55 × 42 μm after protargol staining), the appearance of cortical platelets (vs. without), shape of macronucleus (variable vs. globular), the size of macronucleus (19 × 11 vs. 22 × 17 μm), and having more buccal membranelles (7–10 vs. 6–7), fewer dikinetids in the ventral kinety (4–8 vs. 15–17), and a shorter ventral kinety (6 vs. 28 μm measured from the line drawing in Figure 10.5G).

*Strombidium parasulcatum* n. sp. can be separated from *S. sulcatum* (Granda and Montagnes, 2003) by its larger cell width (30–40 vs. 23 μm *in vivo*), the disappearance of apical protrusion after protargol staining (vs. appearance with different staining methods), its larger buccal cavity width (15 vs. 7 μm), the shape of macronucleus (variable vs. bilobed), the arrangement of extrusomes (no-grouped vs. several in bundle), its shorter ventral kinety (4–8 μm vs. 10 μm), the position of the ventral kinety (occupying posterior 1/4 of cell length vs. posterior 1/3 of cell length), and different numbers of dikinetids in the girdle and ventral kineties (48–64 vs. 37–50 and 4–8 vs. 9–11, respectively).

With reference to the general appearance and even the morphology characters commonly used for species distinguishing of genus *Strombidium*, it is difficult to separate the new species from *S. sulcatum* sensu Song et al. (2000) and Zhang et al. (2010). For example, they are similar in cell size, the appearance of anterior protrusion *in vivo* and after stained, number of collar and buccal membranelles, number of dikinetids of girdle and ventral kinety, and length of ventral kinety. However, the low similarity of the 18S rRNA gene sequences between them demonstrates that they cannot be conspecific. Moreover, their dissimilarities in terms of the position of the largest cell width (equatorial area vs. posterior of mid-body), the width and depth of buccal cavity (15 vs. 7 μm and 25 vs. 33% of cell length), arrangement of extrusomes (no-grouped vs. several in bundle), the position of girdle kinety (posterior one-third vs. mid-body), and the length of cilia in collar membranelles (25 vs. 20 μm) justify naming our organism as a separate species.

### Establishment of Cryptic Species

Given that the high morphology similarities with its congeners, *S. parasulcatum* could be regarded as a cryptic species. The discrimination of cryptic species (morphologically indistinguishable forms that appear to be genetically distinct) has become an urgent issue for taxonomists. To resolve this issue, previous studies suggested that the subtle morphological or ecological differences among the species are required, so we can understand their biogeography and ecological niche differentiation (McManus et al., 2010). Moreover, the ultrastructure and cyst morphology should also be considered (Gruber et al., 2019). However, our study showed that the molecular data could play an important part in the resolution of the cryptic species, as suggested by Santoferrara and McManus (2017).

### Comparison With Similar Congeners’ Growth Rates

Some of oligotrich ciliates have the ability to consume bacteria (Rivier et al., 1985; Bernard and Rassoulzadegan, 1990). Previous studies showed that *S. sulcatum* can graze on pico-sized prey, including live bacteria, heat-killed bacteria, fluorescently labeled bacteria (FLB), and non-living, bacteria-size particles (Fenchel and Jonsson, 1988; Allali et al., 1994; Dolan and Šimek, 1997; Christaki et al., 1998; Dolan, 2018). Neglect of the first 1-day growth rate with negative value in the present study, the growth rate of *S. parasulcatum* grazing on bacteria at the second 24 h cultivation was lower than that of *S. sulcatum* observed by Rivier et al. (1985) in 24 h incubation (about 1.06 vs. 1.56–2.33 day^–1^), although it showed its highest 1-day specific growth rates beginning at day 5 (1.79 day^–1^). The findings demonstrate that both congeners have different trophodynamic parameters.

### Comments on the Phylogenetic Analysis

Five congeners are closely related to *S. parasulcatum* according to the phylogenetic tree (Figure 1). Although *S. parasulcatum* groups with *S. crassulum*, *S. pseudostylifer*, and *S. stylifer* (MP, 100%; BI, 1.00), it is morphologically distinct from these three species (Table 3), i.e., *S. parasulcatum* differs from *S. stylifer* and *S. pseudostylifer* in the absence (vs. presence) of an elongated process/spine and can be distinguished from *S. crassulum* by the different numbers of dikinetids in the girdle and ventral kinety (48–64 vs. 115 and 4–8 vs. 9–15, respectively). *Strombidium parasulcatum* has high similarity with *S. sulcatum* and *S. inclinatum* in morphology, but they do not cluster together in the phylogenetic tree, and their 18S rRNA gene sequences show high dissimilarity compared with *S. pseudostylifer*, *S. stylifer*, and *S. crassulum*. All six congeners belong to the same clade, so more gene sequences and other gene markers are needed in order to resolve the phylogenetic relationships among these taxa and to identity morphological synapomorphies.

## Taxonomic Summary

Class Oligotrichea Bütschli, 1889
Subclass Oligotrichia Bütschli, 1889
Order Oligotrichida Bütschli, 1889
Family Strombidiidae Fauré-Fremiet, 1970
Genus *Strombidium* Claparède and Lachmann, 1859

*Strombidium parasulcatum* n. sp.

*Diagnosis.* Cell 40–50 × 30–40 μm *in vivo*, slightly variable in shape, generally broadly obconical to ellipsoidal with apical protrusion. Macronucleus variable, spherical to ellipsoidal. Adoral zone split into 15 collar and 9 buccal membranelles. Girdle kinety equatorial, continuous, composed of about 55 dikinetids. Ventral kinety extending meridionally in posterior one-fourth of cell ventral, composed of about seven dikinetids.

*Type locality.* Coastal waters off Keelung (25° 08′ 30″ N; 121° 47′ 42″ E), Taiwan.

*Type material.* The holotype with protargol-impregnated specimen is deposited in the Biodiversity Research Center, Academia Sinica, Taipei, Taiwan, ROC (ASIZ01000005). The paratype specimens are deposited in the Natural History Museum, London, United Kingdom (NHMUK 2014.1.8.1).

*Gene sequence.* The accession number for the 18S rRNA gene sequence of *Strombidium parasulcatum* in the GenBank database is KJ101609.

*Etymology.* From *para*, Greek for beside, combined with *sulcatum*. Neuter gender.

*Zoobank registration.* urn:lsid:zoobank.org:pub:428672C2-A57E-46E9-BBEC-EF2669B85FF7.

## Data Availability Statement

The datasets presented in this study can be found in online repositories. The names of the repository/repositories and accession number(s) can be found below: NCBI (accession: KJ101609).

## Author Contributions

S-FT and K-PC conceived the research. S-FT, K-PC, and M-LL conducted the analysis and drafted the manuscript. All authors contributed to the article and approved the submitted version.

## Conflict of Interest

The authors declare that the research was conducted in the absence of any commercial or financial relationships that could be construed as a potential conflict of interest.

## Publisher’s Note

All claims expressed in this article are solely those of the authors and do not necessarily represent those of their affiliated organizations, or those of the publisher, the editors and the reviewers. Any product that may be evaluated in this article, or claim that may be made by its manufacturer, is not guaranteed or endorsed by the publisher.
