# Supplementary material for: Morphological, Molecular, and Growth Characteristics of a Cryptic Species, Strombidium parasulcatum n. sp. (Alveolata: Ciliophora: Oligotrichida)
Source: Front Microbiol. 2022 Feb 10;12:770768. doi: 10.3389/fmicb.2021.770768 (PMC8866572; doi:10.3389/fmicb.2021.770768)
Supplement: Supplementary file 1 [file Data_Sheet_1.docx]

A

B

C

Supplementary 2. **(A)** Abundance of bacteria during the 45-day culture period. (B) Growth curve of *S. parasulcatum* n. sp. during the 45-day culture period. The bars represent the abundance of *S. parasulcatum* over culture time. The solid line represents the abundance of *S. parasulcatum* in log over culture time. The dashed line denotes the maximum growth rate of *S. parasulcatum*. **(C)** Specific growth rates of *S. parasulcatum* n. sp. measured over 1 day (solid line) or 2 days (dashed line) during the 45-day period of culture.
