# Supplementary material for: Morphological, Molecular, and Growth Characteristics of a Cryptic Species, Strombidium parasulcatum n. sp. (Alveolata: Ciliophora: Oligotrichida)
Source: Front Microbiol. 2022 Feb 10;12:770768. doi: 10.3389/fmicb.2021.770768 (PMC8866572; doi:10.3389/fmicb.2021.770768)
Supplement: Supplementary file 2 [file Table_1.DOCX]

Supplementary 1. The oral primordium (arrow in each photo), posterior to the girdle kinety and left of the ventral kinety, was observed in live observation (A-C) and after protargol staining (D-E).
